# Supplementary material for: Genome-Wide Analysis of UGT Genes in Petunia and Identification of PhUGT51 Involved in the Regulation of Salt Resistance
Source: Plants (Basel). 2022 Sep 19;11(18):2434. doi: 10.3390/plants11182434 (PMC9506063; doi:10.3390/plants11182434)
Supplement: Supplementary file 1 [file plants-11-02434-s001.zip › Table S1.pdf]

Table S1. Protein Information of PhUGT.

Accession number	Gene name	Number of amino acid (aa)	Molecular weight (kDa)	Theoretical pI	Instability index	Aliphatic index	Subcellular localization
Peinf101Scf00445g13007.1	PhUGT1	452	51.41	5.35	38.49	94.2	Microsome
Peinf101Scf00364g04006.1	PhUGT2	502	55.50	5.75	48.78	92.03	Microsome
Peinf101Scf01858g01005.1	PhUGT3	492	54.66	5.55	42.8	96.24	Cytoplasm
Peinf101Scf01217g00013.1	PhUGT4	517	58.30	5.53	44.32	86.73	Cell membrane
Peinf101Scf00546g06024.1	PhUGT5	515	57.73	6.81	38.35	92.12	Cell membrane
Peinf101Scf00221g16009.1	PhUGT6	487	54.28	5.68	44.94	95.07	Microsome
Peinf101Scf00920g01024.1	PhUGT7	349	38.98	4.69	43.26	99.94	Microsome
Peinf101Scf00920g00001.1	PhUGT8	483	54.09	5.36	42.59	85.36	Microsome
Peinf101Scf00714g05008.1	PhUGT9	497	56.22	6.15	53.1	91.83	Microsome
Peinf101Scf00920g00003.1	PhUGT10	488	54.14	5.07	51.61	95.08	Microsome
Peinf101Scf00244g11013.1	PhUGT11	475	54.11	5.48	48.63	86.36	Microsome
Peinf101Scf00930g02010.1	PhUGT12	478	53.45	5.85	46.36	98.7	Microsome
Peinf101Scf00471g09012.1	PhUGT13	481	53.54	5.99	41.45	89.11	Microsome
Peinf101Scf00184g02016.1	PhUGT14	473	54.34	5.55	46.17	89.05	Microsome
Peinf101Scf00421g00001.1	PhUGT15	475	53.57	9.37	47.47	86.36	Microsome
Peinf101Scf00122g02020.1	PhUGT16	478	53.10	5.75	39.58	93.39	Microsome
Peinf101Scf00409g06004.1	PhUGT17	473	53.15	5.81	56.32	87.61	Microsome
Peinf101Scf00248g04008.1	PhUGT18	463	52.07	5.64	42.95	88.36	Microsome
Peinf101Scf00821g02023.1	PhUGT19	448	50.52	5.18	43.53	89.62	Microsome
Peinf101Scf01538g02020.1	PhUGT20	493	54.72	4.83	43.26	96.29	Microsome
Peinf101Scf00872g05003.1	PhUGT21	470	52.70	6.08	48.95	88.26	Microsome
Peinf101Scf01074g00004.1	PhUGT22	468	53.16	5.66	42.25	89.72	Cell membrane
Peinf101Scf00170g04016.1	PhUGT23	335	37.39	5.71	33.73	92.51	Cytoplasm
Peinf101Scf01602g01003.1	PhUGT24	467	51.44	5.88	42.62	100.62	Microsome
Peinf101Scf01205g03036.1	PhUGT25	460	51.83	5.13	43.01	92.8	Microsome
Peinf101Scf01117g05001.1	PhUGT26	467	52.76	5.84	49.02	86.34	Microsome
Peinf101Scf00777g02005.1	PhUGT27	467	52.77	5.99	63.82	76.62	Microsome
Peinf101Scf00645g07001.1	PhUGT28	473	53.17	6.52	51.43	92.71	Microsome
Peinf101Scf04354g00002.1	PhUGT29	494	55.84	5.32	34.01	93.6	Microsome
Peinf101Scf02029g00029.1	PhUGT30	309	35.40	6.51	50.26	97.48	Microsome

Peinf101Scf01117g09015.1	PhUGT31	360	41.15	9.21	51.2	89.39	Cytoplasm
Peinf101Scf06781g00003.1	PhUGT32	360	40.66	8.27	44.67	88.97	Microsome
Peinf101Scf00796g00026.1	PhUGT33	478	54.40	6.24	38.13	96.03	Microsome
Peinf101Scf00487g12016.1	PhUGT34	476	52.59	5.95	37.96	102.73	Microsome
Peinf101Scf00457g08001.1	PhUGT35	500	56.01	5.7	36.69	97.3	Microsome
Peinf101Scf01178g03005.1	PhUGT36	504	56.92	5.89	53.38	96.17	Microsome
Peinf101Scf02861g00015.1	PhUGT37	488	55.21	5.81	47.64	93.85	Microsome
Peinf101Scf00267g11001.1	PhUGT38	482	54.46	6.68	44.07	94.54	Microsome
Peinf101Scf00625g16001.1	PhUGT39	482	54.45	6.55	42.53	97.01	Microsome
Peinf101Scf00920g01020.1	PhUGT40	448	50.45	5.19	43.34	93.42	Cytoplasm
Peinf101Scf02472g00001.1	PhUGT41	502	56.74	5.97	38.12	89.1	Microsome
Peinf101Scf00920g00002.1	PhUGT42	422	46.80	5.27	45.43	89.41	Microsome
Peinf101Scf00373g36008.1	PhUGT43	387	44.06	6.33	41.34	93.41	Microsome
Peinf101Scf01107g00001.1	PhUGT44	504	57.01	5.37	40.87	89.27	Microsome
Peinf101Scf00837g10001.1	PhUGT45	488	55.04	5.85	42.19	92.46	Microsome
Peinf101Scf00445g11017.1	PhUGT46	484	56.06	6.96	40.99	94.83	Microsome
Peinf101Scf02339g08002.1	PhUGT47	437	49.49	6.11	49.51	91.44	Microsome
Peinf101Scf00889g05004.1	PhUGT48	368	42.07	6.36	46.91	88.64	Microsome
Peinf101Scf01436g03019.1	PhUGT49	457	50.98	5.73	47.96	88.14	Microsome
Peinf101Scf00086g03003.1	PhUGT50	448	49.57	6.06	38.58	93.35	Microsome
Peinf101Scf01317g03035.1	PhUGT51	466	52.89	5.55	46.39	88.84	Endoplasmic reticulum
Peinf101Scf01353g00005.1	PhUGT52	444	49.94	5.3	43.6	96.8	Microsome
Peinf101Scf01117g08002.1	PhUGT53	447	50.91	5.95	52.59	87.63	Microsome
Peinf101Scf00034g05010.1	PhUGT54	337	38.32	8.71	34.78	103.47	Microsome
Peinf101Scf00250g02011.1	PhUGT55	462	51.62	4.97	45.84	86.28	Cytoplasm
Peinf101Scf01380g08048.1	PhUGT56	467	52.81	5.3	37.44	86	Microsome
Peinf101Scf00019g00004.1	PhUGT57	488	54.34	5.18	42.25	90.45	Microsome
Peinf101Scf00318g14021.1	PhUGT58	433	48.18	5.18	42.17	97.74	Microsome
Peinf101Scf02621g01010.1	PhUGT59	454	51.49	5.91	50.92	91.23	Microsome
Peinf101Scf15053g00004.1	PhUGT60	548	61.54	6.52	46.52	76.99	Microsome
Peinf101Scf00536g01021.1	PhUGT61	332	36.62	5.42	39.95	84.52	Cytoplasm
Peinf101Scf01295g00014.1	PhUGT62	413	46.80	5.63	46.4	83.34	Microsome

Peinf101Scf00777g01016.1	PhUGT63	451	49.02	5.4	39.13	90.8	Microsome
Peinf101Scf01353g00003.1	PhUGT64	460	52.48	5.8	38.28	96.61	Microsome
Peinf101Scf01889g07014.1	PhUGT65	441	49.25	5.2	47.65	86.85	Microsome
Peinf101Scf01393g01001.1	PhUGT66	451	51.35	5.87	39.81	94.24	Microsome
Peinf101Scf00542g01001.1	PhUGT67	463	51.55	6.62	44.31	97.3	Microsome
Peinf101Scf00838g05001.1	PhUGT68	456	51.81	5.35	46.99	96.64	Microsome
Peinf101Scf08918g00005.1	PhUGT69	441	49.57	5.77	47.71	86.64	Microsome
Peinf101Scf00445g13011.1	PhUGT70	434	49.33	6.19	51.73	91.13	Microsome
Peinf101Scf00871g04013.1	PhUGT71	474	53.59	5.41	34.46	89.22	Microsome
Peinf101Scf01294g07004.1	PhUGT72	451	50.99	5.77	40.08	92.71	Microsome
Peinf101Scf01436g08002.1	PhUGT73	452	51.76	8.61	44.94	95.02	Microsome
Peinf101Scf05389g00003.1	PhUGT74	510	57.75	6.26	47.07	86.96	Microsome
Peinf101Scf00140g27001.1	PhUGT75	461	52.12	6.34	58.05	92.62	Microsome
Peinf101Scf02279g03005.1	PhUGT76	451	50.85	7.19	34.58	90.11	Microsome
Peinf101Scf00384g03002.1	PhUGT77	455	51.33	5.09	44.85	91.05	Microsome
Peinf101Scf00206g02001.1	PhUGT78	439	49.56	5.19	43.01	92.32	Microsome
Peinf101Scf01214g00005.1	PhUGT79	573	64.47	5.58	41.9	93.39	Microsome
Peinf101Scf01353g01020.1	PhUGT80	405	46.14	6.52	42.79	96.25	Microsome
Peinf101Scf00100g07011.1	PhUGT81	462	51.92	6.02	45.91	97.06	Microsome
Peinf101Scf01480g04001.1	PhUGT82	453	51.31	5.65	39.69	96.36	Microsome
Peinf101Scf00049g02009.1	PhUGT83	510	56.89	5.84	37.16	98.88	Microsome
Peinf101Scf00982g03005.1	PhUGT84	473	52.67	5.21	44.61	85.16	Microsome
Peinf101Scf01109g08011.1	PhUGT85	423	47.50	5.64	51.56	85.51	Cytoplasm
Peinf101Scf03062g00003.1	PhUGT86	463	52.24	8.62	54.18	88.42	Microsome
Peinf101Scf01969g01029.1	PhUGT87	460	51.29	5.52	40.77	91.78	Microsome
Peinf101Scf01064g05003.1	PhUGT88	463	51.91	5.64	37.45	95.55	Cytoplasm
Peinf101Scf17737g00004.1	PhUGT89	464	52.73	5.98	50.58	80.24	Microsome
Peinf101Scf00765g04002.1	PhUGT90	402	45.48	5.97	45.46	96.69	Microsome
Peinf101Scf00170g06003.1	PhUGT91	403	45.00	5.18	39.45	94.81	Microsome
Peinf101Scf00796g01039.1	PhUGT92	384	43.28	5.16	50.02	95.99	Microsome
Peinf101Scf00459g02001.1	PhUGT93	438	48.91	5.28	40.9	87.85	Microsome
Peinf101Scf01021g02001.1	PhUGT94	470	52.68	5.14	46.77	94.23	Microsome
Peinf101Scf00413g09031.1	PhUGT95	458	51.90	6.28	36.89	88.76	Microsome

Peinf101Scf02793g00017.1	PhUGT96	552	62.32	6.93	50.14	88.33	Microsome
Peinf101Scf01262g04007.1	PhUGT97	468	51.93	5.59	31.78	98.44	Microsome
Peinf101Scf00457g08002.1	PhUGT98	496	55.26	5.42	41.7	101.61	Microsome
Peinf101Scf00170g05019.1	PhUGT99	471	52.22	6.8	37.96	96.26	Microsome
Peinf101Scf00920g01006.1	PhUGT100	479	53.42	5.72	44.05	89.71	Microsome
Peinf101Scf00604g03002.1	PhUGT101	489	55.50	5.41	49.51	99.24	Microsome
Peinf101Scf00999g02027.1	PhUGT102	486	55.08	5.43	41.12	88.21	Microsome
Peinf101Scf00118g07015.1	PhUGT103	506	56.94	6.33	48.57	86.5	Microsome
Peinf101Scf01271g08005.1	PhUGT104	486	54.12	5.39	36.31	94.07	Microsome
Peinf101Scf00920g01023.1	PhUGT105	529	59.19	5.82	42.96	93.42	Microsome
Peinf101Scf02361g01006.1	PhUGT106	298	33.89	6.76	52.21	88.32	Cytoplasm
Peinf101Scf00791g08026.1	PhUGT107	485	54.48	5.93	42.12	91.81	Microsome
Peinf101Scf00545g08008.1	PhUGT108	486	53.02	6.12	42.14	77.24	Microsome
Peinf101Scf00871g02013.1	PhUGT109	480	54.33	5.49	31	89.54	Microsome
Peinf101Scf00652g13002.1	PhUGT110	476	53.36	6.19	51.12	92.35	Microsome
Peinf101Scf00364g03001.1	PhUGT111	466	52.10	5.74	43.79	89.29	Microsome
Peinf101Scf00652g13003.1	PhUGT112	476	53.55	6.13	48.48	92.16	Microsome
Peinf101Scf02055g00016.1	PhUGT113	474	53.79	5.65	45.73	87.17	Microsome
Peinf101Scf00631g01006.1	PhUGT114	323	36.15	5.28	47.97	95.36	Microsome
Peinf101Scf01050g07016.1	PhUGT115	453	51.22	6.21	41.07	87.81	Microsome
Peinf101Scf00622g05007.1	PhUGT116	481	54.43	5.84	30.82	95.99	Microsome
Peinf101Scf00791g00003.1	PhUGT117	468	52.71	6.15	49.8	94.36	Microsome
Peinf101Scf00914g05017.1	PhUGT118	504	56.84	5.45	48.66	94.6	Microsome
Peinf101Scf00405g07004.1	PhUGT119	474	53.66	6.62	44.94	94.37	Microsome
Peinf101Scf01201g00015.1	PhUGT120	475	53.98	6.25	44.19	90.08	Microsome
Peinf101Scf01353g00004.1	PhUGT121	350	39.50	4.87	42.61	93.8	Microsome
Peinf101Scf01117g09007.1	PhUGT122	311	35.34	6.62	46.44	84.63	Microsome
Peinf101Scf00765g03001.1	PhUGT123	308	34.58	5.84	35.64	97.99	Cytoplasm
Peinf101Scf01556g05036.1	PhUGT124	438	48.43	5.66	40.71	91.21	Microsome
Peinf101Scf00073g14003.1	PhUGT125	476	53.81	5.29	35.16	90.29	Microsome
Peinf101Scf00837g06001.1	PhUGT126	469	54.18	5.46	40.17	93.28	Microsome
Peinf101Scf00837g08016.1	PhUGT127	483	54.88	5.91	39.75	87.25	Microsome
Peinf101Scf01775g08003.1	PhUGT128	472	53.93	5.48	47.31	93.14	Microsome

Peinf101Scf01048g01001.1	PhUGT129	471	53.48	5.65	56.76	84.27	Microsome
