# Supplementary material for: Genome-Wide Analysis of UGT Genes in Petunia and Identification of PhUGT51 Involved in the Regulation of Salt Resistance
Source: Plants (Basel). 2022 Sep 19;11(18):2434. doi: 10.3390/plants11182434 (PMC9506063; doi:10.3390/plants11182434)
Supplement: Supplementary file 1 [file plants-11-02434-s001.zip › Table S2.pdf]

Table S2: Primer Sequences in This Study

Primer Name	Primer Sequence
UGT51-qRT-F	TCCATGCATGCACCAACCAACAG
UGT51-qRT-R	TCATAGTAGACAGCAGTAACAGA
UGT51-F	ATGGGAACAGTACAAGAACTATT
UGT51-R	TCACATTGCTGGCTTCTACTTCA
PhGAPDH-F	CAAGGCTGGAATTGCTTTGAG
PhGAPDH-R	CACCACTTTACTCCACTGATGCA
